# 10 recommendations for African governments to ensure food security for poor and vulnerable populations during COVID-19

**DOI:** 10.1007/s12571-020-01062-7

**Published:** 2020-07-12

**Authors:** Laté Lawson-Lartego, Marc J. Cohen

**Affiliations:** Oxfam America, Inclusive and Resilient Food Systems Theme, Washington, DC USA

**Keywords:** Coronavirus, Food security, Nutrition, Sub-Saharan Africa

## Abstract

In addressing COVID-19, African governments should not forget the livelihoods as well as the food and nutrition security of their citizens. With over 70% of the workforce in the informal sector without any social protection and health insurance, the pandemic could have a devastating impact on income and livelihoods as well as food and nutrition security for workers up and down the food chain. There are ten steps African governments can take to ensure that their response to the disease takes food and nutrition security into account:

1. Protect food supply chains and consider them essential services;

2. Consider fiscal and monetary incentives;

3. Prioritize healthy diets;

4. Use food reserves wisely;

5. Keep food markets open while ensuring safety;

6. Use mobile cash transfers for social protection;

7. Protect farmers and food workers;

8. Prioritize gender equality;

9. Instill a sense of solidarity; and

10. Avoid export bans.

“In addition to my fear for health because of the COVID-19 pandemic, I am also concerned about food scarcity and prices skyrocketing. The price of smoked herring which is a basic source of protein we buy from traders from Ghana has doubled overnight as the government is closing down marketplaces as well as borders”. Those are a few words from my (Laté Lawson-Lartego’s) mother in Togo when I spoke to her last, to check on her amidst the COVID-19 spread.

While I applaud governments in Togo and elsewhere on the continent for taking these difficult and devastating — but necessary — measures to slow down COVID-19’s proliferation, they should carefully think through the implications of social distancing for every segment of their population. Moreover, they should analyze how these measures will impact local food systems and the women and men who produce, process, and sell food for their living, who are the majority of the African workforce. In places like Togo and many other African countries, cross border trade for food and other goods are important for daily life. In addition, with over 70% of economically active Africans in the informal sector without any social protection and health insurance, if the lockdown and social distancing are prolonged, this could result in a devastating impact on income and livelihoods as well as food and nutrition security for workers up and down the food chain (Tan 2020). Consumers in cities will not be spared either if food availability is thrown into jeopardy or if prices increase (Resnick 2020).

If innovative solutions are not found and people are worried about what to eat and other basic needs, they will not respect the lockdown and social distancing that are necessary to combat the COVID-19 pandemic. This, in turn, will expose many people in Africa and other developing countries to a greater risk of infections. Are governments in Africa prepared for the worst-case scenario?

Even before the emergence of the novel coronavirus, Sub-Saharan Africa was a global hotspot of hunger and poor health, home to 39% of the world’s severely food insecure people (FAO 2019). It has the highest worldwide prevalence of malaria, tuberculosis, and HIV, with 3324 people per doctor and 985 per nurse/midwife, the least favorable such ratios globally (WHO 2020). According to the African Center for Disease Control (2020), surveys in 16 African countries indicate that only about a third of households can access clean water and soap, with poor and rural families particularly disadvantaged. By mid-June 2020, the region recorded 222,000 cases of COVID-19 (WHO 2020).

The disease is already pushing economies around the world into turmoil. The think tank IFPRI estimates that with the global economy contraction, over 140 million people could be pushed into poverty. This will have direct, negative consequences for hunger and food insecurity (Glauber et al. 2020). In rich countries such as here in the U.S., we see Congress and the government include measures to stimulate the economy, protect people living in or near the poverty line, and protect workers forced to stay home with generous unemployment benefits and other protections. Sadly, in Africa, this won’t be the case as many governments are paralyzed with heavy debt. Many informal workers will have to depend on the solidarity and generosity of their family in-country and in the diaspora.

Concerns about food prices are amplified by a number of analyses by the Food and Agriculture Organization of the United Nations and the World Food Program, many think tanks, and media outlets, and demand thoughtful action. Governments should make sure that food trade is not interrupted, prices remain stable, and incentives are provided to all key players in the food chain to ensure that people, especially the most vulnerable populations — including seniors, children, and students — have access to healthy and nutritious food.

The World Health Organization (2020) reports that many African governments have taken effective public health measures to contain the disease. These include rapid scale up of health personnel and capacities, quarantines, social distancing measures, testing, and contact tracing. By late June, African officials began to think about reopening markets and businesses.

Ten recommendations are listed below for the use of African governments and those of other continents to consider as they deal with COVID-19 and put in place an Emergency Food & Nutrition Security plan for their population. These recommendations are based on years of experience in working on food and nutrition security and food systems issues in a number of crisis situations as well as on well-documented evidence from literature:

1. Protect food supply chains and consider them essential services: While locking down the population may be an adequate measure, and we hear that many governments are imposing curfews among other measures, food supply chains should be considered as essential services as are health, pharmacy, banking, etc. Many governments are doing this and countries in Africa could learn from this action. If borders are closed, governments should allow for essential goods and businesses, including the movement of food, to continue.
2. Consider fiscal and monetary incentives: Governments should also consider setting up incentives such as lifting VAT, duties, and other taxes imposed on food business to enable the supply chain to continue to function properly without any huge interruption. Governments should also consider extending concessional loans or loan guarantee facilities to food actors who may need it to ensure the food supply chain is running smoothly. Some countries in Africa are already considering such measures. To the extent practicable, restaurants and food shops should be allowed to continue to operate where they can maintain physical distancing requirements and explore alternative delivery systems.
3. Beyond calories — prioritize healthy diets: If food prices go up, consumers may resort to changing their consumption habits or purchasing less nutritious foods, which are often cheaper. Governments can help with the accessibility of fresh fruits and vegetables to ensure they remain part of a healthy diet. In addition, governments should also encourage producers of fresh food and provide them the needed incentive to stay in business and access to markets while ensuring their protection and food safety.
4. Use food reserves wisely and target appropriately: Many governments hold grain and other food reserves. Governments should monitor prices and release food from these reserves if prices spike. In addition, governments should also not hesitate to distribute or request assistance from businesses or international and national organizations to distribute food to those in need. Appropriate targeting should be ensured to avoid political exclusion of certain groups and elites. Women heads of households, orphans, widows, refugees, internally displaced people, senior citizens, the poor, and other vulnerable people should be the principal target of food distribution. If possible, school feeding programs should be maintained or modified to enable children who depend on those meals to continue to have access. There are already well-developed mechanisms and a wealth of experience on how to target effectively, and governments should seek out the necessary partnerships and expertise.
5. Keep food markets open while ensuring safety and social distancing: The vast majority of Africans continue to buy their food in open markets. Shutting down those marketplaces should be the last resort. Instead, governments should deploy advice for food safety and ensure social distancing in open markets. A few countries are already coming up with innovative ways of doing this, and governments and municipalities in Africa should learn and apply best practices while keeping their citizens safe and healthy.
6. Use mobile cash transfer and protect the poor and the vulnerable: Governments have the duty to protect the most vulnerable and poor people in society. Given the income lost due to stay at home orders and social distancing measures, governments should consider a minimum monthly cash allocation to their most at risk populations to enable them to put food on the table and cover other basic needs. Again, careful targeting should be an utmost consideration. With the proliferation of mobile money, governments should consider forging strategic partnerships with mobile companies and banks to enable this in the most efficient and secure manner. Poor countries’ governments could use the debt payment relief they are getting from international financial institutions as well as any additional budget support to do this. Of course, they can’t compete with the generous social safety net in rich countries, but this is the time for governments in Africa to put the wellbeing of their populations at the center and give a chance to the pandemic to be overcome. This will provide people with a much-needed financial cushion. In the areas where hunger and food insecurity are prevalent, our experience and the evidence tells us that a good part of the cash assistance households and individuals receive will be spent on purchasing food.
7. Protect farmers and food workers: Farmers and people who provide labor to plant, harvest, transform, and distribute our food are the backbone of our food systems — and should be supported and protected during the pandemic. Women often play a key role in this which is often not well recognized or equally compensated. This segment of the population should be considered a part of the essential function and should be treated well, supported, and protected against the pandemic and any exploitation to avoid disrupting the food system and protect their rights. Governments should introduce special measures to ensure this and for continued service and education. Extension services and access to information, for instance, can now be delivered digitally to farmers without the need for physical human contact. Delivery of subsidized inputs and other support provided by the government to farmers should not be interrupted, but rather, should be done in the safest manner. Other support functions and business along the food chain should also be considered as essential functions and be supported accordingly.
8. Prioritize the needs of women and gender equality: Gender inequality is still rampant in many countries in Africa and this pandemic could exacerbate the problem. When it comes to food and nutrition security, women play a significant role in food production as well as transformation and food preparation. With school closed, women will have an additional burden of care. Governments should sensitize men, boys, and other non-gender binary people to consider splitting responsibility at home. Governments should also ensure that all measures and policies are gender-sensitive and do not further widen the gender gap. Social protection services should also continue to operate and ensure sensitization against gender-based violence, which is at a risk of increasing with the social distancing and shelter-in-place measures.
9. Instill a sense of solidarity in citizens: Governments should empower communities and people to display solidarity and mutual support in these difficult times. While those who are better off will tend to stock up on food and create artificial price gouging, governments should encourage and enforce price control and ensure that every person is able to buy food at a reasonable price. Governments should also incentivize individuals, businesses, and charitable organizations to donate food to community food banks and to the needy. If appropriate, governments should also consider fiscal incentives such as tax deductions for individuals and businesses and other forms of public recognition and celebration of generosity and solidarity.
10. No export bans: Finally, Governments should not take actions, such as export bans, that will drive up global prices and encourage panic buying or hoarding.

We hope these recommendations are useful and will help prevent hunger and food insecurity as we fight this pandemic. Nutritious food is a source of vitality and good health. We are already hearing alarming outcries from citizens in Africa in countries such as Togo where my (Laté Lawson-Lartego’s) mother lives, as well as other countries such as Zimbabwe (Muronzi 2020) and many more. People fear to die from hunger more than COVID-19. Governments should do all they can, in partnership with one another, the private sector, and civil society, to combat this pandemic. The number one emphasis is on strengthened engagement with the private sector to maintain food supplies through supply chains and markets, wherever possible, but safely. They should put their population at the center, ensure that the food sector is considered essential, and instill policies to make sure everyone gets enough to eat and respects all the prevention measures. However, it is important to note that there is limited capacity of humanitarian aid and government safety nets to deal with the far-reaching global consequences of a global pandemic.

## References

[CR1] African Center for Disease Control. (2020). COVID-19 scientific and public health policy update. https://africacdc.org/download/covid-19-scientific-and-public-health-policy-update-16-june-2020/. Accessed 22 June 2020.

[CR2] Food and Agriculture Organizations of the United Nations (FAO) (2019). State of food and nutrition in the world 2019.

[CR3] Glauber, J., Laborde, D., Martin, W., and Vos, R. (2020). Trade restrictions are worst possible response to safeguard food security. IFPRI Blog. https://www.ifpri.org/blog/covid-19-trade-restrictions-are-worst-possible-response-safeguard-food-security. Accessed 1 May 2020.

[CR4] Muronzi, C. (2020). 'We'll die of hunger first': Despair as Zimbabwe lockdown begins. Al-Jazeera, 30 March. https://www.aljazeera.com/news/2020/03/die-hunger-despair-zimbabwe-lockdown-begins-200330054919081.html. Accessed 1 May 2020.

[CR5] Resnick, D. (2020). COVID-19 lockdowns threaten Africa’s vital informal urban food trade. IFPRI Blog. https://www.ifpri.org/blog/covid-19-lockdowns-threaten-africas-vital-informal-urban-food-trade. Accessed 1 May 2020.

[CR6] Tan, H. (2020). A food crisis looms as coronavirus forces farms to stay idle and countries hoard supplies. World Markets. https://www.cnbc.com/2020/03/30/coronavirus-food-crisis-looms-as-farms-idle-countries-hoard-supplies.html. Accessed 1 May 2020.

[CR7] World Health Organization. (2020). Regional Office for Africa Website. https://www.afro.who.int/. Accessed 22 June 2020.

